# Field trial evidence of non-transgenic and transgenic Bt. rice genotypes in north of Iran

**DOI:** 10.1186/s43141-020-00028-8

**Published:** 2020-05-01

**Authors:** Salman Dastan, Behzad Ghareyazie, Shahpour Abdollahi

**Affiliations:** Department of Biosafety and Genetic Engineering, Agricultural Biotechnology Research Institute of Iran (ABRII), Karaj, Iran

**Keywords:** *Bacillus thuringiensis*, *cry*1Ab gene, GM rice, Stem borer, White heads, Yield loss

## Abstract

**Background:**

Field-testing genetically modified crops provides scientists with an opportunity to collect information on environmental interactions and agronomic performance, which is critical to a full environmental safety assessment as required by regulatory authorities. As a result, the goal of this research was field trial of transgenic and conventional rice genotypes. The experiment was carried out in a randomized complete blocks design (RCBD) with four replications and seven genotypes in three isolated regions under the biosafety standard protocol in north of Iran in 2016. In this study, four transgenic lines with an active *cry*1Ab gene in the vegetative stage and three conventional genotypes (control) as treatment were assessed.

**Results:**

The findings demonstrated that in all three regions, transgenic lines derived from Khazar cultivar, were similar to their parent(s) in terms of growth phenology, agronomical traits, grain amylose content, gel consistency, and gelatinization temperature. In all the three regions, the highest number of panicle per m^2^, number of filled spikelet per panicle, and filled spikelet percentage per panicle were obtained for transgenic lines. Khazar cultivar compared to the transgenic lines showed lower paddy yield. In all the three regions, transgenic lines had lower yield loss than their non-transgenic parent. The lowest number of white heads belonged to transgenic Tarom Molaii and transgenic lines. The most positive direct effect on paddy yield was related to the number of filled spikelet per panicle. Thus, this can be a good trait to achieve higher yield derived from reducing the negative indirect effect of dead heart and white heads.

**Conclusion:**

It can be concluded that by producing transgenic rice, which is resistant to stem borer with an active promoter in the reproductive stage, farmers can reduce significant part of yield loss resulting from dead heart and white heads directly correlating with the number of filled spikelet per panicle and paddy yield.

## Background

Rice (*Oryza sativa* L.) is the staple food of more than half of the world’s population and has an obvious effect on feeding, income, and job creation of people in the world, especially in Iran [27]. According to the official statistics released by FAO, the area cultivated with rice in the world during the past years was from 145 million to over 160 million hectares [8]. The last global statistics showed that paddy yield and white rice productions were 742 and 492.2 million tons respectively in 2014, respectively [8]. The same amount was predicted for 2016. Iran has 550,000 ha of paddy field and two million tons of white rice production and has a 0.4% share in rice production and cultivation area in the world, most of which (about 75%) is located in the northern strip, i.e., the provinces of Guilan, Mazandaran, and Golestan, and the remaining (25%) of the paddy field is located in other 13 provinces with different climates [23]. Rice production in the world has been facing serious challenges since 2000 making the international community concerned about food supply for people in the world. As shown by investigations, the most important method to fight rice stem borer (*Chilo suppressalis* Walker) in Iran and also most regions in the world has been the chemical inputs [23]. Therefore, making transgenic rice cultivars by the gene *cry*1Ab of the bacterium Bt. is the best method to give plants resistance against the invasion of pests and to reduce the environmental damage by chemical pesticides [9].

Plant biotechnology has traditionally encompassed the application of cell and tissue culture for crop improvement. Since the mid-1980s, the development and application of transgenic plants has been the dominant research activity associated with plant biotechnology. More recently, research has expanded to include the application of genomics technologies [2]. Field-testing genetically modified crops provides scientists with an opportunity to collect information on environmental interactions and agronomic performance, critical to a full environmental safety assessment as required by regulatory authorities.

To estimate the yield benefit and cost of insect-resistant GM rice, Xia et al. [39] compared field productivity of three insect-resistant rice lines with their non-transgenic parental cultivars. Their findings revealed that great benefits obtained by the yield-related traits were detected in the transgenic lines when high insect pressure was recorded, but a cryptic yield loss was observed when the level of insect pressure was extremely low. In fact, yield loss produced in the transgenic lines was under low insect pressure. A strategic field extension should be required when insect-resistant lines are commercialized to circumvent the unnecessary yield losses; this probably applies to other insect-resistant transgenic crops [39].

Among the agronomic traits of rice, yield is the most important one. It possesses three main components: number of tillers (panicles) per plant, number of grains per panicle, and 1000-grain weight. Yield is a complex quantitative trait; in addition to the three main components, there are also some other traits relevant to rice yield, such as plant height, plant architecture, biomass, and seed-setting rate [18, 24, 35]. Therefore, in the process of improving rice yield trait, we should take multiple agronomic traits into consideration [6]. Klumper and Qaim [21] considered a meta-analysis of the impacts of genetically modified crops. They carried out a meta-analysis of the agronomic and economic impacts of transgenic crops to consolidate the evidence. On average, GM technology adoption has reduced chemical pesticide use by 37%, increased crop yields by 22%, and increased farmer profits by 68%. Yield gains and pesticide reductions are larger for insect-resistant crops than for herbicide-tolerant crops. Yield and profit gains are higher in developing countries than in developed countries [21]. Perhaps great economic and impressive approach to protecting rice against insect attack is to cultivate insect-resistant rice cultivars. Overall, the objectives of this experiment were (1) to examine the quantitative and qualitative parameters of non-transgenic and transgenic rice cultivars, (2) to estimate the pest loss and stem borer resistance parameters including dead heart and whit heads, and (3) to identify genetic correlation and regression model by path analysis.

## Methods

### Description of the regions

Experiments were done in isolated paddy fields in three sites, under the control of Iran Rice Research Institute between Alborz Mountains range and the Caspian Sea in Mazandaran and Guilan provinces in north of Iran (Table 1) in 2016. The geographical coordinates and soil properties of the three sites are shown in Table 1. Solar radiation was estimated using sunshine hours and extraterrestrial radiation [31–33]. For calculating solar radiation, the *Srad_calc* program was used. This program uses the sunshine hour data to calculate solar radiation. For counting day length, the *PP_calc* program was applied. *Srad_calc* and *PP_calc* programs can also be downloaded from “https://sites.google.com/site/CropModeling.”

**Table 1:** Description of the geographical coordinate, soil properties (0–30 cm) and climatic parameters of three rice production sites. As well as description of name, origin, and other characteristics of rice genotypes

Description	Sari (Mazandaran province)		Amol (Mazandaran province)		Rasht (Guilan province)		
Geographical coordinate	36°39′22.52″N, 53°9′42.55″E		36°23′59.24″N, 52°31′37.55″E		37°13′28.78″N, 49°38′57.85″E		
Soil properties	Sari (Mazandaran province)		Amol (Mazandaran province)		Rasht (Guilan province)		
Soil texture	Clay loam		Silt clay		Clay		
EC (dSm^−1^)	0.92		0.96		0.98		
pH	7.25		7.58		6.60		
Organic matter (%)	2.46		2.11		2.31		
Phosphorus (mg kg^−1^)	12.50		8.90		19.60		
Potassium (mg kg^−1^)	185		208		205		
Climatic parameters	Sari (Mazandaran province)		Amol (Mazandaran province)		Rasht (Guilan province)		
	Experiment period	Mean 15 years	Experiment period	Mean 15 years	Experiment period	Mean 15 years	
Minimum temperature (°C)	18.4	18.3	18.9	18.5	13.8	17.6	
Maximum temperature (°C)	28.4	25.2	27.7	26.9	33.6	26.5	
Mean temperature (°C)	23.4	22.8	23.3	32.2	22.3	22.1	
Evaporation (mm)	143.7	147.6	109.5	120.8	99.6	121.4	
Rain (mm)	52.5	89.0	50.8	93.4	77.7	60.7	
Mean humidity (%)	74.7	73.5	75.8	77.5	78.7	78.0	
Mean sunshine hours	221.1	208.8	187.6	182.7	186.2	213.9	
Solar radiation (MJ m^−2^ d^−1^)	19.3	19.5	17.8	17.9	17.7	18.1	
Genotype	Backcross (♀ × ♂)	Description	Plant height	Yield condition	Maturity	Type	Origin
Nemat	Improved	Non-transgenic	Semi-dwarf	High yield	Late maturing	Improved	Iran
Khazar	Improved	Non-transgenic	Semi-dwarf	Medium yield	Medium maturing	Improved	Iran
Tarom-Hashemi	Local	Non-transgenic	Tall	Low yield	Early maturing	Local	Iran
Tarom-Molaii	Local	Transgenic	Tall	Low yield	Early maturing	Local	Iran
KHT_2_	Khazar/Bt. Tarom-Molaii	Transgenic	Semi-dwarf	Medium yield	Medium maturing	Improved	Iran
KHT_3_	Khazar/Bt. Tarom-Molaii	Transgenic	Semi-dwarf	Medium yield	Medium maturing	Improved	Iran
KHT_4_	Khazar/Bt. Tarom-Molaii	Transgenic	Semi-dwarf	Medium yield	Medium maturing	Improved	Iran

### Description of the experiment

**Fig. 1:**
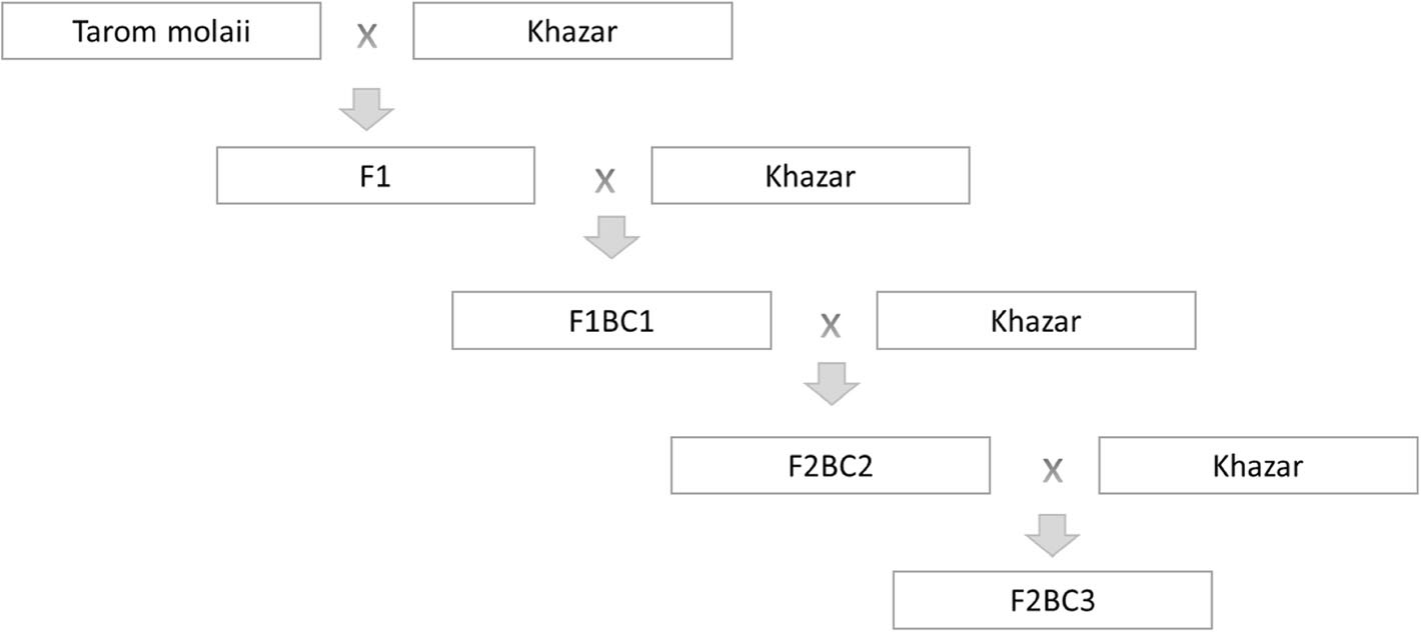
The diagram of breeding scheme of backcross lines

The experiment was carried out in a randomized complete blocks design (RCBD) with four replications. In this experiment, there were three transgenic rice lines including KHT_2_, KHT_3,_ and KHT_4_ (driven from the backcross of Khazar cultivars with the transgenic line of Tarom Molaii) containing the gene *cry*1Ab from the bacterium Bt. (*Bacillus thuringiensis*) with the transgenic line of Tarom Molaii (containing the gene for resistance against striped stem borer as the non-restorer parent) along with the non-transgenic cultivars. Three non-transgenic rice cultivars were Tarom Hashemi, Nemat, and Khazar. In fact, in this experiment breeding and transgenic of cultivars were considered to use genetic potential of Tarom Molaii cultivar. Therefore, in backcross, 12.5% of Tarom Molaii background was maintained and in F_2_. In addition to transgenic selection, selection was made based on superior rice characteristics and morphological traits. The diagram of breeding scheme of backcross lines is shown in Fig. 1. The profile of the genotypes is shown in Table 1.

The isolation conditions were met according to biosafety principles (standard of biosafety protocol) consisting of cultivation with distance from other farmers’ paddy fields. This allowed for putting enough margin space around the field, harvesting the margin separately, and eliminating the plants’ residue after the harvesting. The experiment’s time and space distance were also considered adhering to biosafety principles.

Considering the climates of Mazandaran and Guilan provinces, seedlings were transplanted in 3–4 leaf stages. Considering the type of the cultivar, the transplanting operation was done in all the three regions with similar situations. The size of each plot was decided to be 4 × 7 m^2^, and planting density was 16 plants per square meter by 25 × 25 cm^2^ planting arrangement. Nitrogen, phosphorus, and potassium fertilizers were used in each region according to the suggestions of Iran Rice Research Institute and by considering the result of soil analysis (Table 1). All phosphorus amount and one-third of nitrogen fertilizer were used as basal in paddy field preparation stage. Two-thirds of nitrogen fertilizer was used as top-dressing in panicle initiation and full heading stages. Sixty percent of potassium fertilizer was used as basal and the remaining amount was used as top-dressing in tillering and panicle initiation stages (splitting to 20% in each stage). Depth of irrigation was set at 5 cm according to agricultural principles. In order to control weeds’ growth and the mixing of water and fertilizer in paddy fields, nylon plastic cover was put at the borders to the depth of 30 cm.

Crop protection practices, irrigation, weeding, and fertilization, were done in the isolated experiment paddy field in each region. But, no pesticide was used so that the lines’ resistance could be accurately observed. Nits of stem borer were taken from infected fields and distributed in the experimental fields ultra-optimally. Other crop management practices were done according to the standards of biosafety protocol and Standard Evaluation System (SES) of International Rice Research Institute (IRRI).

### Measurements

#### Agronomical traits

During the growing period, after the removal of marginal effect in each site, all agronomical traits (phenological traits, morphological traits, and yield components) were randomly measured according to Standard Evaluation System (SES) of International Rice Research Institute (IRRI). Paddy yield, straw yield, and biological yield were measured by harvesting hills from 4 m^2^ in the middle part of each plot based on 12% of moisture. From the relationship between paddy yield and biological yield, the harvest index was calculated and expressed in percentage.

The grain filling period was determined based on the time between pollination and physiological maturity (50% of the panicles turned yellowish green). The average of grain filling rate in the unit of area was obtained by dividing the paddy yield based on the length of grain filling period according to g m^−2^ d^−1^. According to the difference in growing pattern and cultivars’ maturity, the thermal index of GDD was calculated for grain filling period from pollination to physiological maturity stages according to the formula below:

GDD = *T*_max_ + *T*_min_/*T*_b_ × 100

Where *T*_max_ is maximum daily temperature, *T*_min_ is minimum daily temperature, and *T*_b_ is based temperature. The temperatures below 10 and above 30 °C were considered 10 and 30, respectively.

In order to calculate leaf area index the during pollination stage, the maximum length and width for all leaves in ten hills per plant were measured in centimeters, and it was estimated by the formula LA = 0.75 × LW, where LA is leaf area, and L and W are the leaf’s length and width, respectively.

### Determination of grain quality traits

Measuring baking qualitative traits, such as grain amylose content, gelatinization temperature, gel consistency, grain length, grain width, grain elongation, baked grain length, baked grain width, baked grain elongation, conversion efficiency, and healthy and broken grain percentage was done by Standard Evaluation System (SES) of International Rice Research Institute (IRRI).

### Estimation of yield loss

For estimating yield loss, Islam and Karim’s [15] method was used. In this method, in the white head stage, 16 plants were chosen per experimental plot. In every plant, the measured number of healthy panicle (*N*), the number of fairly healthy panicle (*N*´), and the number of blank panicle (*N*´´) were measured. The number of healthy spikelet per panicle (*P*), the number of fairly healthy spikelet per panicle (*P*´), and total paddy yield (*Y*) were measured randomly by 16 plants per plot.

$$ \mathrm{Y}\mathrm{ield}\kern0.17em \mathrm{loss}\left(\%\right)=\frac{\left[\mathrm{P}/\mathrm{N}\left(\mathrm{N}\acute{\mkern6mu}+\mathrm{N}\acute{\mkern6mu}\acute{\mkern6mu}\right)\right]}{P+P\acute{\mkern6mu}}\kern0.5em \times \mathrm{Y} $$

### Evaluation of pest loss

Sorting of infected percentage of plants for determining sensitive and resistance lines was evaluated by Heinrichs [14] method. In order to determine the dead heart, 30, 50, and 65 days after transplanting, and in order to determine the white heads, sampling was done a week before the harvest. In every sampling from each plot, 10 hills per plant were randomly selected, and the number of plants with dead hearts (DH) related to the damage from the insect’s first generation, and the number of white heads (WH) related to the damage from the insect’s second generation were counted and their percentages were calculated. Infected percentage of plants was measured by Gomez and Gomez [10] through the formula below:

$$ \mathrm{D}.\mathrm{H}/\mathrm{W}.\mathrm{H}\left(\%\right)=\frac{\mathrm{Number}\ \mathrm{of}\ \mathrm{infected}\ \mathrm{stem}\mathrm{s}\ast \mathrm{Number}\ \mathrm{of}\ \mathrm{infected}\ \mathrm{plants}}{\mathrm{Total}\ \mathrm{measured}\ \mathrm{plants}\ast \mathrm{Number}\ \mathrm{of}\ \mathrm{stem}\ \mathrm{per}\ \mathrm{infected}\ \mathrm{plants}}\kern0.5em \ast 100\kern1em $$

### Statistical analysis

After normalization, data analyzed by the SAS statistical software and averages’ comparison were calculated by LSD tests in a 5% probability level. To determine those traits that had the most effect on yield, after applying regression to eliminate alignment, path analysis was performed by genotype correlation with the Path software.

## Results

### Combined mean square analysis of investigated traits

Findings of the compound analysis variance in Table 2 showed that all investigated traits including phenological traits, morphological traits, yield components, quantitative yield and harvest index, yield loss, dead heart, and white heads were significant in 1% probability level on genotype treatment. DT, DF, DFM, SL, FS, SY, BY, HI, and YL were significant in 5% probability level under the effect of regions; also DFM, PL, TH, FTH, ITH, PM, FSP, BS, BSP, TGW, PY, DH, and WH were significant in 1% probability level on region. At interaction of region × genotype, DFM, FLL, FTP, ITH, ITP, and HI were significant statistically in 1% probability level. FLA, LAI, FTH, PM, YL, and WH were significant in 5% probability level under the interaction of region and genotypes (Table 2).

**Table 2:** Combined mean square and mean comparison of different traits of rice genotypes in three sites

Traits	Mean square				Mean comparison							
	Location (L)	Genotype (G)	L × G	C.V. (%)	Genotype							
DF	2	6	12	–	Nemat	Khazar	Hashemi	Molaii	KHT_2_	KHT_3_	KHT_4_	LSD 0.05
Days from transplanting to tillering (DT)	*	**	NS	5.16	60.67b	55.67c	51.50c	45.92d	60.00ab	62.50a	62.42a	2.41
Days from transplanting to 50% of flowering (DF)	*	**	NS	3.81	94.42c	89.67d	86.17e	80.67f	95.33bc	98.33a	97.50ab	2.86
Days from transplanting to full maturity (DFM)	**	**	**	3.58	121.83ab	115.33c	111.33d	109.92d	120.58b	124.17a	123.47ab	3.46
Filling period (FP)	*	**	NS	15.77	16.83a	16.83a	15.42a	13.33b	16.25a	15.50a	13.25b	1.98
Filling rate (FR) (g m^−2^ d^−1^)	NS	**	NS	19.18	353.70bc	263.69d	302.04 cd	271.54d	369.71b	385.51b	474.89a	54.29
GDD in pollination stage	NS	**	NS	16.05	271.17a	272.42a	243.58ab	208.33c	261.58a	249.17a	213.42bc	32.27
Plant height (PH) (cm)	NS	**	NS	5.77	119.75b	119.83b	133.83a	130.92a	122.00b	122.25b	119.58b	5.86
Stem length (SL) (cm)	*	**	NS	6.34	93.26b	93.04b	106.38a	104.85a	97.34b	97.93b	95.35b	5.34
Panicle length (PL) (cm)	**	**	NS	8.13	26.49a	26.79a	27.45a	26.07ab	26.66bc	24.53bc	24.23c	1.71
Flag leaf length (FLL) (cm)	NS	**	**	8.61	27.18c	34.07ab	32.71b	35.68a	26.82c	26.19c	26.17c	2.10
Flag leaf width (FLW) (cm)	NS	*	NS	13.45	1.12ab	1.13a	1.02abc	0.95c	1.01abc	1.06abc	0.95c	0.11
Flag leaf area (FLA) (cm)	NS	**	*	15.57	22.46bc	28.36a	24.92b	25.10b	20.01 cd	20.55 cd	18.48d	2.91
Leaf area index (LAI)	NS	**	*	15.56	2.25bc	2.84a	2.49b	2.51b	2.00 cd	2.06 cd	1.85d	0.29
Number of tiller per hill (TH)	**	**	NS	9.25	17.33b	15.83c	14.75c	15.67c	18.50ab	18.92a	18.92a	1.30
Number of fertile tiller per hill (FTH)	**	**	*	11.78	10.92b	10.33bc	9.42c	11.25b	14.00a	15.08a	14.50a	1.18
Fertile tiller percentage per hill (FTP)	NS	**	**	6.56	63.03d	65.31d	64.05d	71.16c	75.58b	79.85a	76.52ab	3.80
Number of infertile tiller per hill (ITH)	**	**	**	17.77	6.42a	5.50b	5.33b	5.42c	4.50c	3.83c	4.42c	0.72
Infertile tiller percentage per hill (ITP)	NS	**	**	15.86	36.92a	34.79a	35.88a	28.92b	24.43c	20.27d	23.53 cd	3.80
Number of panicle per m^2^ (PM)	**	**	*	9.18	277.58b	252.58c	237.00c	250.58c	295.83ab	301.83a	301.58a	20.58
Number of spikelet per panicle (SP)	NS	**	NS	9.28	108.75a	106.33a	94.08b	79.25c	102.50a	101.50ab	103.67a	7.55
Number of filled spikelet per panicle (FS)	*	**	NS	10.77	86.83a	83.92a	73.92b	65.17c	87.42a	89.08a	89.50a	7.25
Filled spikelet percentage per panicle (FSP)	**	**	NS	3.47	79.79d	78.68d	78.44d	82.33c	85.39b	87.98a	86.16ab	2.35
Number of blank spikelet per panicle (BS)	**	**	NS	13.26	21.92ab	22.42a	20.17b	14.08 cd	15.08c	12.42d	14.17 cd	1.87
Blank spikelet percentage per panicle (BSP)	**	**	NS	16.36	20.21a	21.34a	21.44a	17.70b	14.60c	12.05d	13.77 cd	2.32
Thousand grain weight (TGW) (gr)	**	*	NS	5.11	24.79a	23.42b	23.31b	23.29b	23.89ab	23.58b	23.78b	0.99
Paddy yield (PY) (kg ha^−1^)	**	**	NS	8.26	5838a	4391b	4520b	3579c	5929a	5924a	5949a	349.94
Straw yield (SY) (kg ha^−1^)	*	**	NS	11.11	6228c	5599d	7051b	5939 cd	7699a	7494ab	7489ab	616.90
Biological yield (BY) (kg ha^−1^)	*	**	NS	9.28	12066b	9990c	11571b	9518c	13628a	13418a	13438a	907.79
Harvest index (HI) (kg ha^−1^)	*	**	**	4.11	48.46a	44.04b	39.25c	37.67d	43.54b	44.25b	44.28b	1.45
Yield loss (YL) (kg ha^−1^)	*	**	*	25.33	412.33ab	428.42a	340.75c	218.42d	313.58c	359.08abc	341.83bc	71.50
Dead heart (DH)	**	**	NS	9.73	0.16bc	0.24ab	0.27a	0.00d	0.11c	0.11c	0.06 cd	0.11
White heads (WH)	**	**	*	29.35	2.79bc	4.34a	3.44b	3.00b	2.11 cd	2.29 cd	2.06d	0.69

ns, *, and **: non-significant and significant in 5% and 1% probability level, respectively

*Values within a column followed by same letter are not significantly different at LSD (*P* ≤ 0.05)

### Mean comparison analysis of investigated traits

According to the findings, the vegetative and reproductive periods in Rasht region were higher than two other sites (Table 2). The lowest vegetative and reproductive growth periods of cultivars were observed in Sari region. Nemat cultivar and transgenic lines derived from Khazar cultivar were long-term in terms of vegetative and reproductive growth period, and Khazar cultivar was in the next rank. Two local cultivars, Tarom Hashemi and Tarom Molaii, stood in third ranks. Attending to interaction of location and genotypes, we can find out in the three sites that the highest DFM was observed for non-transgenic Nemat cultivar and transgenic lines including KHT_2_, KHT_3,_ and KHT_4_. In the three sites, the least growth period was obtained for local cultivars, including Tarom Hashemi and Tarom Molaii (Table 3).

**Table 3:** Mean comparison of days to full maturity, flag leaf length, flag leaf area, flag leaf index, tillers parameters, number of panicle per m^2^, yield loss, harvest index, and white heads of rice genotypes in three sites by slice interaction

Interaction (traits group I)	Days to full maturity (DFM)			Flag leaf length (cm)			Flag leaf area (cm)			Flag leaf index		
	Sari	Amol	Rasht	Sari	Amol	Rasht	Sari	Amol	Rasht	Sari	Amol	Rasht
Nemat	121.00a	121.75a	122.75a	27.50b	27.10ab	26.95b	22.93bc	22.11ab	22.33b	2.30ab	2.21ab	2.23ab
Khazar	114.50ab	114.75b	116.75ab	34.20a	31.95a	36.05a	32.66a	24.51a	27.90a	3.27a	2.45a	2.79a
Tarom Hashemi	108.00b	111.50b	114.50ab	28.40b	33.13a	36.60a	26.40b	26.96a	21.40bc	2.64ab	2.70a	2.14ab
Tarom Molaii	101.75b	108.75a	109.25b	35.60a	34.90a	36.55a	24.35bc	25.41a	25.56ab	2.44ab	2.54a	2.56a
KHT_2_	118.25a	119.75a	123.75a	27.73b	28.18ab	24.55b	17.72c	22.93ab	19.38bc	1.77b	2.29ab	1.94b
KHT_3_	122.25a	121.75a	128.50a	27.20b	26.63b	24.75b	17.82c	21.68b	22.15b	1.78b	2.17b	2.22ab
KHT_4_	119.00a	119.50a	131.75a	28.40b	26.15b	23.95b	16.43c	20.07b	18.94c	1.64b	2.01b	1.89b
Interaction (traits group II)	Number of fertile tiller per hill			Fertile tiller percentage per hill			Number of infertile tiller per hill			Infertile tiller percentage per hill		
	Sari	Amol	Rasht	Sari	Amol	Rasht	Sari	Amol	Rasht	Sari	Amol	Rasht
Nemat	10.00b	11.50b	11.25b	60.02b	66.49b	62.60c	6.75a	5.75a	6.75a	39.99a	33.51a	37.25a
Khazar	10.75b	11.75b	8.50c	62.79b	69.10b	64.03c	6.50a	5.25a	4.75ab	37.21a	30.90a	36.25a
Tarom Hashemi	10.00b	9.75c	8.50c	59.69b	67.19b	65.27c	6.75a	4.75b	4.50ab	40.31a	32.81a	34.50ab
Tarom Molaii	14.00a	11.00b	8.75c	77.83a	79.91ab	63.74c	4.00b	4.25b	5.00ab	22.18b	28.09b	36.50a
KHT_2_	15.00a	14.75a	12.25ab	75.90a	75.56ab	75.28b	4.75ab	4.75b	4.00b	24.10b	24.44bc	24.75b
KHT_3_	15.25a	16.00a	14.00a	77.08a	78.85a	83.61a	4.50ab	4.25b	2.75c	22.92b	21.15c	16.75c
KHT_4_	15.00a	15.50a	13.00a	76.52a	75.41ab	77.63b	4.50ab	5.00b	3.75bc	23.49b	24.59bc	22.50b
Interaction (traits group III)	Number of panicle per m^2^			Yield loss (kg ha^−1^)			Harvest index (%)			White heads (%)		
	Sari	Amol	Rasht	Sari	Amol	Rasht	Sari	Amol	Rasht	Sari	Amol	Rasht
Nemat	268b	277b	288a	435.50a	472.50a	329.00b	50.25a	46.75b	48.38a	3.28ab	3.21ab	1.88bc
Khazar	276b	270b	212b	371.75b	470.75a	442.75a	45.50b	43.75bc	42.88bc	4.71a	4.18a	4.14a
Tarom Hashemi	268b	235c	208b	370.50b	357.75c	294.00c	41.25bc	37.25c	39.25c	3.52ab	4.63a	2.17b
Tarom Molaii	288b	244c	220b	222.25d	274.25e	158.75d	38.75c	36.00c	38.25c	3.85ab	3.57ab	1.59c
KHT_2_	316a	312a	260a	270.00cd	332.00d	338.75b	42.75bc	44.25bc	43.63bc	2.32b	1.79b	2.23b
KHT_3_	316a	322a	268a	419.00a	342.00cd	316.25bc	43.00bc	43.50bc	46.25b	2.20b	2.80ab	1.87bc
KHT_4_	312a	325a	269a	293.50c	402.50b	329.50b	42.00bc	45.25bc	45.60b	2.45b	1.89b	1.85bc

*Values within a column followed by same letter are not significantly different at LSD (*P* ≤ 0.05)

The highest and lowest FP was observed for all cultivars in Rasht and Sari. GDD in pollination stage varies in different regions, indicating that temperature variations vary the overall rice growth period. Mean comparison of genotypes showed that the maximum grain FP was related to the two KHT_2_ and KHT_3_ lines, Nemat, Khazar and Tarom Hashemi cultivars. Nemat and Khazar cultivars along with KHT_2_ and KHT_3_ lines had the highest GDD in pollination stage. The lowest grain FP and GDD in pollination stage was obtained for Tarom Molaii cultivar, but the least grain FR was related to Khazar cultivar (Table 2). Genetic factors (cultivar) somewhat determine the grain FR, and environmental factors (temperature) determine the extent of grain FP. It seems that grain FR of most cultivars was higher due to PM. When number of panicle per of unit area is higher, it causes faster source depletion (leaf and stem).

Mean comparison of treatments showed that among genotypes, Tarom Hashemi and Tarom Molaii had the highest PL and SL. Among semi-dwarf cultivars, the lowest PL and SL belonged to Nemat, Khazar and transgenic lines derived from Khazar. The highest LAI belonged to Khazar cultivar, the main cause of which was the higher FLA of this cultivar (Table 2). Mean comparison by slice interaction showed that in all the three regions, the highest FLL belonged to Khazar, Tarom Hashemi, and Tarom Molaii cultivars. In all the three regions, transgenic lines derived from Khazar cultivar were similar to their parents in terms of FLL and FLA. In all the three areas, the highest LAI belonged to Khazar cultivar. Nemat, Tarom Hashemi, and Tarom Molaii cultivars with a lower difference stood in later ranks. In all the three regions, the lowest LAI belonged to Khazar transgenic lines (Table 3). Mean comparison shows differences between the three regions in terms of these traits due to the climatic conditions and soil properties of these regions. Genotypes mean comparison showed that the highest PM belonged to KHT_3_ and KHT_4_ lines. The low PM in Tarom Molaii cultivar has its genetic potential, which has lower degree of tiller production than modified cultivars (Table 2).

According to mean comparison, using slice interaction transgenic lines had higher FTH, fertile tiller percentage, lower ITH, and ITP compared to non-transgenic cultivars (Table 3). In all the three regions, mean comparison by slice interaction method showed that the highest PM was obtained for transgenic lines derived from Khazar cultivar. Also, in Rasht region, Nemat cultivar along with Khazar transgenic lines showed the highest PM. Local cultivars, Tarom Hashemi and Tarom Molaii, showed the lowest PM (Table 3).

Genotype mean comparison showed that the highest paddy yield belonged to Nemat cultivar and transgenic lines derived from Khazar cultivar. Khazar cultivar compared to the transgenic lines, KHT_2_, KHT_3_, and KHT_4_, showed 25.49, 25.88, and 19.26% lower paddy yield, respectively (Table 2). The lowest paddy yield was attributed to two local cultivars, Tarom Hashemi (4520 kg ha^−1^) and Tarom Molaii (3579 kg ha^−1^). In terms of straw yield and biological yield, cultivars had a high variation (Table 2). Mean comparison by slice interaction method showed that in all the three regions, transgenic lines derived from Khazar had higher harvest index than their non-transgenic parent. Also, in all the three regions, the lowest harvest index was observed for Tarom Molaii cultivar (Table 3).

### Qualitative traits

According to findings, there were no significant differences between transgenic lines and their parents in terms of technological traits and grain baking related traits. In terms of grain length before and after baking, the transgenic lines KHT_2_, KHT_3_, and KHT_4_ were similar to Khazar cultivar. In fact, the coherence in the shape and paddy yield is the first factor in the quality corroboration. Farmers consider these factors for the cultivation of new cultivars (Table 4).

**Table 4:** Technological and qualities traits of rice genotypes in three sites. As well as rice genotypes ranking by Heinrichs method for dead heart and white heads

Genotype	Grain length before baking (mm)	Grain width before baking (mm)	Grain shape	Grain length after baking (mm)	Grain elongation (mm)	Conversion efficiency (%)	Healthy grain (%)	Broken grain (%)	Amylose content (%)	Gel consistency (mm)	Gelatinization temperature	Dead heart percentage		White heads percentage	
												Rate	Rank	Rate	Rank
Nemat	8.26	1.93	4.30	13.73	1.64	65.00	57.00	8.00	27.50	39.67	7.00	16bc	Medium resistance	27.90cd	Sensitive
Khazar	7.07	1.84	3.85	11.20	1.58	62.51	55.82	6.69	22.10	56.67	4.40	24ab	Low resistance	43.40a	Sensitive
Hashemi	7.29	1.86	3.97	12.53	1.69	62.04	54.10	7.94	21.50	44.00	3.47	27a	Low resistance	34.40b	Sensitive
Molaii	6.50	1.88	3.45	13.01	1.71	68.30	48.40	19.90	21.20	41.85	4.20	0e	Resistant (no damage)	30.00bc	Sensitive
KHT_2_	7.60	1.96	3.87	12.39	1.63	75.80	66.47	9.33	24.50	58.00	3.80	11cd	Medium resistance	21.10e	Relatively sensitive
KHT_3_	7.76	1.88	4.12	12.34	1.59	75.07	68.80	6.27	25.20	58.00	4.60	11cd	Medium resistance	22.90de	Relatively sensitive
KHT_4_	8.00	1.93	4.14	12.40	1.55	74.13	61.86	12.27	27.60	57.40	3.30	6de	Relatively resistant	20.60e	Relatively sensitive
Min.	6.50	1.84	3.45	11.20	1.58	62.04	48.40	6.27	21.20	39.67	3.30	0	–	20.6	–
Mean	7.50	1.90	3.96	12.51	1.63	68.98	58.92	10.04	24.23	50.80	4.40	13.57	–	28.61	–
Max.	8.26	1.96	4.30	13.73	1.71	75.80	68.80	12.27	27.60	58.00	7.00	27	–	43.40	–
SD	0.6	0.04	0.28	0.76	0.06	6.01	7.19	4.78	2.71	8.48	1.24	3.62	–	3.12	–
SE	0.23	0.01	0.10	0.29	0.02	2.27	2.72	1.81	1.03	3.21	0.47	70.53	–	28.81	–

The grain appearance is important to retain its quality and market-friendliness. Grain length before baking is one of the qualitative factors of rice grain. This trait was not influenced by transgenic lines. Transgenic lines and their conventional parent have no significance in terms of grain length after baking and grain elongation (Table 4). In terms of grain conversion efficiency and healthy grain percentage, transgenic lines have a better condition compared to non-transgenic parents. Rice grains increase after baking, and if this increase in length is without increase in thickness, it can be a positive factor for cultivars. There were no significant differences between transgenic lines and their parents in terms of grain amylose content, gel consistency, and gelatinization temperature (Table 4).

### Yield loss (YL), dead heart (DH), and white heads (WH)

Based on the results, the highest YL was observed for Khazar cultivar and Nemat cultivar. Yield loss of transgenic lines derived from Khazar cultivar was significantly lower than their non-transgenic parent. Also, the lowest YL was obtained for Tarom Molalii (Table 2). Mean comparison by slice interaction method showed that in all the three regions, transgenic lines derived from Khazar had lower YL than their non-transgenic parent. In all the three regions, the lowest YL was observed for Tarom Molaii cultivar (Table 3).

Differences in dead heart and white heads in the three regions were related to pest variation in each region. Genotype mean comparison showed that the highest and lowest dead heart and white head rates belonged to Tarom Hashemi and Tarom Molaii. The dead heart rate of Khazar transgenic lines was statistically lower than their non-transgenic parent. The lowest WH belonged to KHT_4_ (Table 2). Mean comparison by slice interaction method showed that in all the three regions, the white heads belonged to Khazar cultivar. Tarom Hashemi and Nemat ranked second with fewer variations. The lowest number of white heads belonged to transgenic Tarom Molaii cultivar and transgenic lines derived from Khazar cultivar, which were diverse in terms of numerical diversity (Table 3).

### Stem borer resistance traits

The results of rice genotypes ranking based on dead heart percentage and white heads percentage are presented in Table 4. In terms of dead heart, Tarom Molaii cultivar suffered no damage and was recognized as a resistant cultivar. KHT_4_ line got placed in group I (relatively resistant), whose percentage of infection was below 10%. KHT_2_ and KHT_3_ lines and Nemat cultivar got placed in group III (medium resistance), where dead heart was below 20%. Tarom Hashemi and Khazar cultivars stood in group V (low resistance), where dead heart was equal to 24% and 17% (Table 4).

### Correlation analysis

**Fig. 2:**
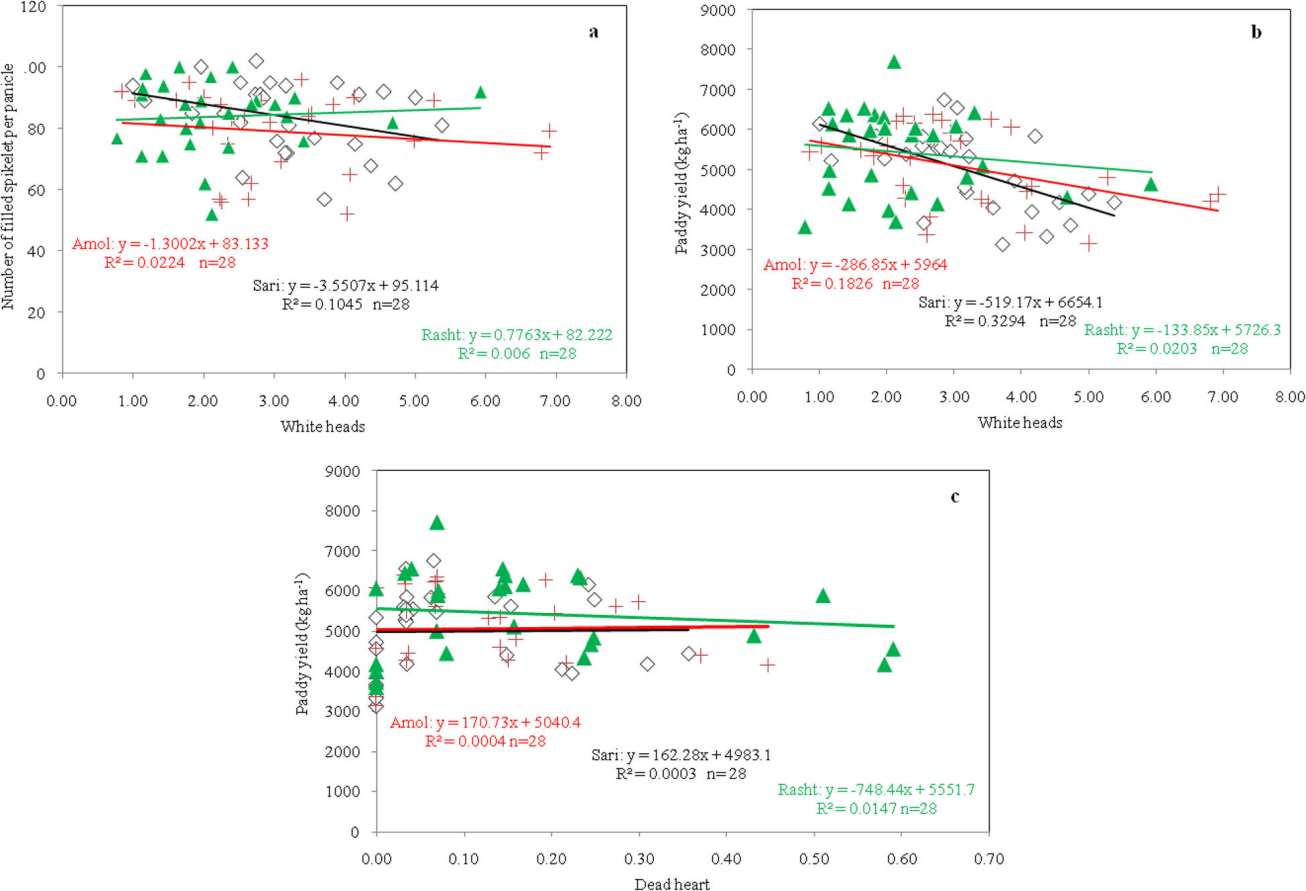
Relation between filled spikelet per panicle with white heads (**a**), paddy yield with white heads (**b**), and paddy yield with dead heart (**c**) of rice genotypes in three sites

According to findings, there was a negative correlation between FS and white heads in the three sites (Fig. 2a). We observed negative correlations between paddy yield with dead heart and white heads in the three sites (Fig. 2b, c). Negative correlation between these traits showed that with the increase of dead heart and white heads, FS and paddy yield had decreased. This can be a hindrance to aggregating the high value of these variables in a genotype, especially if this negative relationship is genetic forms and due to genetic connectivity.

### Path coefficient analysis

Path analysis was used to determine the direct and indirect effects of the variables introduced in the stepwise regression model in the three regions (Table 5). Therefore, genetic correlation was used for the estimation of direct and indirect traits on paddy yield. The most positive direct effect on PY was about FS per panicle. The total direct and indirect effects of this trait on PY (genetic correlation with paddy yield) were equal to 0.93. The indirect effect of FS per panicle by DF, PM, and HI was 1.179, 1.126, and 1.139, respectively (Table 5). According to the direct effect of FS on PY, its indirect effect by DF and PM can be a proper trait to achieve higher yield which is obtained by reducing the negative indirect effect of DH and WH. The maximum negative indirect effect regarding FS per panicle was achieved by PH and WH (Table 5). However, PM has a negative direct effect on PY; but it has a positive indirect effect on FS, which can be considered as a useful trait for selecting higher yield and used in indirect selection discussion (Table 5). The most negative indirect effect on paddy yield is about PM and WH. The sum of direct and indirect effects of these traits was 0.91 and − 0.78, respectively. The indirect effect of PM by all investigated traits was obtained to be more than one. Indirect effect of WH was positive by PM, FS, and HI; also, it has negative effects by DF, PH, ITH, and DH (Table 5).

**Table 5:** Amount of direct and indirect effect of entered traits of rice genotypes to production model by genetic correlation

Traits	Direct effect	Indirect effect by traits								Correlation with paddy yield
		DFM	PH	ITP	PM	FS	HI	DH	WH	
Days to full maturity (DFM)	0.036		− 0.028	− 0.018	0.033	0.032	0.031	0.013	0.028	− 0.94^**^
Plant height (PH)	− 0.17	0.127		− 0.038	0.125	0.145	0.135	− 0.033	− 0.068	− 0.71^**^
Infertile tiller percentage (FTH)	− 0.121	0.059	− 0.027		0.083	0.039	0.008	− 0.081	− 0.083	− 0.42^ns^
Panicle per m^2^ (PM)	− 1.472	− 1.355	1.089	1.015		− 1.252	− 1.031	0.794	1.28	0.91^**^
Filled spikelet per panicle (FS)	1.324	1.179	− 1.14	− 0.438	1.126		1.139	− 0.12	− 0.756	0.93^**^
Harvest index (HI)	− 0.062	− 0.054	0.049	0.004	− 0.043	− 0.05		0.009	0.03	0.83^**^
Dead heart (DH)	− 0.005	− 0.002	− 0.001	− 0.004	0.002	0	0		− 0.004	− 0.23^ns^
White heads (WH)	− 1.212	− 0.933	− 0.824	− 0.824	1.053	0.69	0.605	− 0.812		− 0.78^**^
Remain effect										0.275

## Discussion

In all the three regions, transgenic lines derived from Khazar cultivar were similar to their parent in terms of phenological growing. According to other researchers’ findings, phenology is one of the major topics in the field of ecology, and its aim is to study of changes in the vital stages of plants. Determining the vital stages of plants, including the evaluation of phenological traits, to improve yield and help to make decisions to maximize available plant sources, is very important [41]. The phenology and growth period of rice is one of the main factors determining the agronomical and ecological suitability of cultivars in the cultivated area [41]. Therefore, proper prediction of phenological stages of crops is important for optimizing management activities in the field and better adaptation of crop schedule [16, 19]. In fact, phenological developmental stages in plants are affected by temperature, photoperiod, and vernalization [11, 34]. Adaptation of the growth period of cultivars to achieve acceptable yields, increase productivity, reduce losses, and maintain their cultivation is always one of the important and influential topics. Therefore, the development of medium and late cultivars cultivation in paddy fields in the northwestern part of Iran is highly dependent on the crop rotational calendar, especially seeding date in nursery, seedling age, transplanting date, and growth period of rice cultivars. In fact, flag leaf, with its location close to the panicles, has a major contribution to the transfer of photosynthetic material and grain filling. Hence, in order to achieve maximum yield, the amount of leaf in the shoots of the plant is essential. Davatgar et al. [4] revealed that most of the reduction in leaf area appears to be the consequence of revealed cell expansion, the closing of stomata, and inhibition of photosynthesis.

Genotype mean comparison showed that transgenic lines derived from Khazar cultivar compared to their non-transgenic parent had a higher number and percentage of FS with lower number and percentage of ITH. The most TGW was observed in Nemat cultivar. The TGW weight belonged to Khazar cultivar and its transgenic lines and local cultivars, Tarom Hashemi and Tarom Molaii, all of which were on a same statistical level. The studied trait variation in different regions was due to the effects of environmental conditions (soil and climate) and other factors (planting and harvesting dates). Yield components are affected by management practices, genotypes, and environment and are often used to justify the cause of yield reducing or increasing. Also, an ecosystem affects the ability of a plant to exhibit genetic potential. In addition, incorrect crop management, water usage, nutrition, temperature, light, and other inappropriate environmental factors can reduce one or more components of the yield components. Also, the number of spikelet per panicle depends on genetic ability of cultivars, climatic conditions at panicle formation, pollination, size, and activity of photosynthetic system at panicle formation period and photosynthetic material transferring capacity to panicle and competition between individual plants. One thousand-grain weight is a genetic trait and is less affected by environmental conditions. This trait depends on the size and duration of the activation of carbon dioxide fixation in plant shoots, photosynthetic material capacities loading to grain, duration of grain formation, weather conditions, mineral amount in grain filling period, and pests and diseases’ occurrence.

In order to overcome the issue of low productive tillers, some researchers have proposed new plant type rice with low-tillering and large panicle size [20]. Among rice tillers, the panicle development pattern is hierarchical and grain yields decrease for each successive tiller [28]. In such circumstances, the yield contribution of the inferior tillers could not be ignored because they possess high productivity potential in theory, as the totipotency of rice coleoptile tissues [26]. If the limiting factors between the inferior and superior tillers could be reduced, or even removed, the yields of the inferior tillers might be dramatically improved. In addition, high-tillering rice cultivars possess a good capacity for functional compensation, and late emerging tillers may suitably compensate for yield losses when nascent tillers are subjected to environmental stresses [38]. Studying the influence of tiller heterogeneity on yield components of rice grown under a different nitrogen treatment, Wang et al. [37] indicated that the quantitative proportions and yield components were decreased in superior tillers and increased in the inferior tillers. The present study suggested that the enhancement of grain filling and weight of the inferior tillers would be an impressive approach to further improve rice yield per acre [37]. Panicle is one of the important traits for rice plant type because it can directly affect grain yield as a factor of sink size [42]. To increase the value of each panicle, an effective strategy to improve grain’s function should be adopted [6]. Previous researchers have indicated that grain weight was the most stable yield component, which was a highly heritable trait less affected by environmental factors [13].

The difference between paddy yield and biomass was due to environmental conditions (climate and soil) and pests and diseases variation in each region. In fact, the formation of vegetation that has a high performance depends on controlling components that interfere with plant growth and development. Achieving the highest yield in transgenic cultivars was the result of providing optimal growth conditions. Therefore, yield loss due to damage of stem borer in transgenic cultivars was much lower than non-transgenic cultivars. Paddy yield of Nemat and Khazar cultivars and transgenic lines derived from Khazar was higher than Tarom Hashemi and Tarom Molaii cultivars. Because Nemat and Khazar cultivars are dwarf and there is the small distance between source and sink the majority of photosynthetic materials are transferred to grain. The semi-dwarf characteristic of these cultivars reduces the competition between vegetative and reproductive organs for photosynthetic materials. Their paddy yield was higher in comparison with two tall cultivars Tarom Hashemi and Tarom Molaii. This is because in tall cultivars, distance between source and sink is high, and most energy and nutrition that plays a decisive role in the transfer of photosynthetic materials to grain is used for vegetative growth. Remobilization of photosynthetic materials in rice is such that in the pollination and heading stages, produced photosynthesis material is more than the need for these two processes. The excess of photosynthetic material is transferred to stem and stored as starch, and in grain filling stage, starch materials are converted to sugar and transferred to these grains. Therefore, because of the small distance between source and sink, semi-dwarf cultivars are more likely to transfer nutrition supply to grain and have more success compared to tall cultivars. By field trials of two fertilizer levels and 18 modern cultivars on the paddy yield of rice, Li et al. [22] revealed that variation among cultivars had a great effect on paddy yield. Close correlations were observed between paddy yield and effective panicles and dry matter production. Harvest index, the ratio of grain weight to total shoot weight, is an important trait associated with the dramatic increases in crop yields. Most progress in improving harvest index occurred following the introduction of semi-dwarf traits into rice in the 1960s [30]. However, the scope for continued harvest index increases in modern rice cultivars was limited by the need for maintaining sufficient leaf area and stem biomass for interception of solar radiation, physical support, and storage of assimilates and nitrogen used in grain filling [3]. Jianchang et al. [17] revealed that post-anthesis dry matter production of high-yield rice differs among different yield categories.

Rice baking traits are mainly determined by the properties of starch, which comprises up to 90% of endosperm of white rice and consists of two amylose and amylopectin components. The high amylose rice is completely separated after drying and hardened after cooling. Most rice with lower amylose content has spikelet with a minimum amount of blank grain and number of broken grains [36]. Measurement of gel consistency complements the amylose test, which indicates the amount of baked rice gel movement. Gel consistency shows the exact hardness and adhesion of cooked rice. Determination of endosperm starch gelatinization temperature is an important test for determining the quality of baking in rice. The temperature of the gelatinized starch is an important quality factor that is related to the baking time and the nature of baked rice; in fact, the higher the gelatinization temperature, the higher the ranking is in the qualitative categorization. Studying effects of genotype and environment on bread making quality in wheat, Dencic et al. [5] showed that cultivar and environment interaction had significant effect on all quality traits. Also, variances of quality traits associated with genetic factors (cultivar) were generally larger than those for cultivar by environmental interaction effects.

In terms of white heads, transgenic lines derived from Khazar cultivar got placed in group 7 (relatively sensitive) which had white heads between 16–25%. Other cultivars stood in group 9 (sensitive); it means that the percentage of white heads of these cultivars was above 25%. By increasing the number of larvae in stem, their survival rate in the stem increases and yield decreases in the same way. Also, the reduction of tillers per hill increases the probability of stem damage. However, if there are several tillers, the tillers are not contaminated will produce the crop, and plants can recover from the damage caused by pests. By comparing three transgenic rice lines, Moghaieb [25] reported that on transgenic rice, no surviving larvae were recorded, and a larval mortality of up to 100% was found 4 days after infestation with stem borers. Conversely, on control rice cultivars, all the recovered larvae were observed healthy without any larval mortality. Moreover, the transgenic rice was very toxic to the rice stem borer (*Chilo agamemnon*) under laboratory condition [25].

The correlation of traits can be due to genes’ connection or the existence of a genetic interaction with an environmental component. Ye et al. [40] also did not observe significant correlations between harvest index and grain yield among modern wheat varieties. With relatively little possibility for increases in grain yield by improving harvest index, greater yield potential must come from increases in net primary productivity [3]. Li et al. [22] revealed that it was important to increase net dry matter production over the entire growing season to achieve a high grain yield. It was consistent with the opinion of Donmez et al. [7].

Path coefficient analysis revealed that direct selection for number of fertile spikelet per panicle and harvest index would likely be effective for increasing grain yield [29]. Direct selection of number of effective tillers per plant, grain yield per plant, hundred grain weight, grain breadth, grain length, and grain thickness would increase harvest index. This study also indicated that there is no common causal factor directly influencing both grain yield and harvest index, though one hundred grains’ weight, grain length, grain breadth, and grain thickness could be augmented in selection criteria for the simultaneous improvement of both the traits [29]. Path coefficient analysis in Gravois and Helms [12] studies revealed that panicle density had the largest positive direct effect on rice yield. Even under low rates of seeding, the number of filled grain per panicle increased, which compensated for the reduced panicles density. Direct effects for filled grain per panicle and grain weight were of secondary and/or tertiary importance in determining rice yield. Unfilled grain per panicle had negligible effects on rice yield. To achieve optimum rice yields and grain quality in a direct-seeded cultural system, adequate panicle density per unit area of uniform maturity must be achieved. Path analysis for paddy yield indicated that the number of spikelet per panicle and flag leaf length had positive direct effects and days to complete maturity, and plant height had negative direct effects on paddy yield under optimum irrigation condition, while flag leaf width and number of filled grains per panicle had positive direct effects, and days to 50% flowering had negative direct effect on paddy yield under drought stress condition [1].

## Conclusion

According to combined variance analysis, there were significant differences among genotypes for all studied traits. According to results of mean comparison, the existence of minimum and maximum values for different traits in the same applied conditions for genotypes in each region indicates genetic variation. This means that by plant breeding, we can improve that trait. Therefore, this issue can provide a basis for determining the best cultivar according to the conditions of the region for cultivation. To determine the direct and indirect effects of the variables entered into the stepwise regression model, path analysis was used. The most positive direct effect on paddy yield was related to number of filled spikelet per panicle. The total direct and indirect effects of this trait on paddy yield (genetic correlation with paddy yield) were 93%. Regarding the direct effect of number of filled panicle per panicle on paddy yield and its indirect effect by day to 50% flowering and number of panicle per square meter, this trait can be a proper one for achieving higher yields, achieved by reducing the negative indirect effect of dead heart and white heads.

## Data Availability

The datasets used and/or analyzed during the current study are available from the corresponding author on reasonable request.

## Abbreviations

GMO: Genetically modified organisms
GDD: Growth-degree-day
